# Vermittlung digitaler Kompetenzen in der Pflegeausbildung – eine Vergleichsanalyse der Rahmenpläne von Bund und Ländern

**DOI:** 10.1007/s00103-022-03575-2

**Published:** 2022-08-09

**Authors:** Sebastian Hofstetter, Lisa Lehmann, Max Zilezinski, Jenny-Victoria Steindorff, Patrick Jahn, Denny Paulicke

**Affiliations:** 1 Universitätsmedizin Halle (Saale), AG Versorgungsforschung|Pflege im Krankenhaus, Department für Innere Medizin, Medizinische Fakultät, Martin-Luther-Universität Halle-Wittenberg, Ernst-Grube-Str. 40, 06120 Halle (Saale), Deutschland; 2 Digital HealthCare Hub, Dorothea-Erxleben-Lernzentrum Halle (DELH), Halle (Saale), Deutschland; 3 Philosophische Fakultät I, Orientalisches Institut, Seminar für Japanologie, Martin-Luther-Universität Halle-Wittenberg, Dachritzstr. 12, 06108 Halle (Saale), Deutschland; 4 Philosophische Fakultät II, Institut für Musik, Medien- und Sprechwissenschaften (IMMS) Abteilung Sprechwissenschaft und Phonetik, Martin-Luther-Universität Halle-Wittenberg, Emil-Abderhalden-Str. 26–27, 06108 Halle (Saale), Deutschland; 5 Medizinische Fakultät, Dorothea-Erxleben-Lernzentrum (DELH), Martin-Luther-Universität Halle-Wittenberg, Deutschland Magdeburger Str. 12, 06112 Halle (Saale),; 6 Fachbereich Medizinpädagogik, Akkon Hochschule für Humanwissenschaften, Berlin, Deutschland

**Keywords:** Pflegeberufereform, Digitale Ausbildung, Pflegeexpert:innen, Professionalisierung, Digitale Transformation, Nursing profession reform, Digital education, Advanced practice nursing, Professional development, Digital transformation

## Abstract

**Hintergrund und Ziel:**

Die digitale Transformation der Gesundheitsversorgung erfordert auch in den Pflegeberufen veränderte Kompetenzen. Die Reform der Pflegeausbildung eröffnet die Chance, die dafür notwendigen Inhalte in der Berufsausbildung zu verankern. Die „Rahmenpläne der Fachkommission nach § 53 Pflegeberufegesetz“ bilden für die Bundesländer die Grundlage zur Erstellung eigener Rahmenlehr- und Ausbildungspläne. Die vorliegende Arbeit untersucht, in welchem Umfang und in welcher Form die Rahmenpläne Digitalisierung aufgreifen.

**Material und Methoden:**

Die Rahmenpläne wurden zwischen August und Oktober 2021 einer explikativ-qualitativen Inhaltsanalyse unterzogen. Dabei wurde zunächst die Häufigkeit von zuvor definierten Schlagwörtern festgestellt. Im Anschluss erfolgte eine systematische Kontextanalyse.

**Ergebnisse:**

Lediglich 6 Bundesländer hatten einen eigenen Rahmenplan erstellt, die anderen verwendeten den Bundesrahmenplan, der nur in geringem Umfang auf den Kompetenzerwerb im Bereich Digitalisierung eingeht. In den eigenen Rahmenplänen der Bundesländer wurde Digitalisierung in unterschiedlichem Maß thematisiert, jedoch insgesamt nur punktuell. Handlungsempfehlungen für praktische Übungsformate wurden kaum gegeben.

**Diskussion:**

Der Erwerb von Kompetenzen im Bereich Digitalisierung bildet das Fundament für das spätere Berufsleben und ist ein wichtiger Bestandteil der digitalen Transformation. Im Rahmen der Modifizierungsmöglichkeit der Pflegeausbildung bis zum Jahr 2024 sollte das Thema noch stärker fokussiert werden. Auch direkt an den Fach- und Berufsschulen sowie Hochschulen kann nachgebessert werden, da die Rahmenpläne teilweise lediglich einen empfehlenden Charakter haben.

**Zusatzmaterial online:**

Zusätzliche Informationen sind in der Online-Version dieses Artikels (10.1007/s00103-022-03575-2) enthalten.

## Abstract (en)

**Background and objective:**

The digital transformation of healthcare requires changed competences in the nursing professions. The reform of nursing education opens up the opportunity to anchor the requisite content in vocational education. The framework curricula of the expert commission (“Rahmenpläne der Fachkommission nach § 53 Pflegeberufegesetz”) form the basis for the federal states to create their own framework curricula. This paper examines to what extent and in what form the framework curricula take up digitalisation.

**Material and methods:**

The framework curricula were investigated in an explicative-qualitative content analysis between August and October 2021. First, the frequency of previously defined keywords was determined. This was followed by a systematic context analysis.

**Results:**

Merely six federal states had created their own framework curriculum; the others used the federal framework curriculum, which only addresses the acquisition of competences in the field of digitalisation to a small extent. Digitalisation was addressed to varying degrees in the federal stateʼs own framework plans but only selectively overall. Recommendations for practical exercise formats were hardly given.

**Discussion:**

The acquisition of competences in the area of digitalisation forms the foundation for later professional life and is an important component of the digital transformation. In the context of the possibility of modifying nursing education until 2024, the topic should be taken into focus more strongly. Improvements can also be made directly at technical and vocational schools as well as universities since the framework curricula are sometimes only of a recommendatory nature.

## Hintergrund

Die unter dem Schlagwort „digitale Transformation“ diskutierten Veränderungsprozesse setzen politische, gesellschaftliche und ökonomische Akteure weltweit unter Handlungsdruck, da die Auswirkungen auf Wirtschaft, Politik und Gesellschaft(en) vielfältig und multidimensional sind [1, 2]. Die japanische Regierung spricht beispielsweise in diesem Zusammenhang bereits vom neuen globalen Zeitalter der „Society 5.0“ [3], womit für Japan die Vision einer auf den Menschen ausgerichteten Gesellschaftsform beschrieben ist, die sowohl ökonomische als auch soziale Belange durch eine Vernetzung des virtuellen Cyberraumes und der physischen Lebenswelt (realer Raum) integriert. Smarte Technologien wie künstliche Intelligenz (KI), Robotik, Internet of Things (IoT), aber auch Augmented (AR) und Virtual Reality (VR) oder Robotic Process Automation (RPA) sollen in Japan dazu beitragen, eine intelligente, besser vernetzte und nachhaltige Gesellschaft zu schaffen [3–5]. Es wird die dahinterliegende Erwartung deutlich, dass mit einer fortschreitenden Digitalisierung auch eine Verbesserung der Lebenswelt und eine Steigerung der Lebensqualität assoziiert sind [6]. Auch in Deutschland ist Digitalisierung als politische Forderung parteienübergreifend Thema [7]. Dabei wird bemängelt, dass Deutschland in Sachen Digitalisierung großen Nachholbedarf habe und hinsichtlich einer digitalen Modernisierung anderen Nationen nachstehe [7]. Gleichzeitig vollzieht sich die digitale Transformation aber „unausweichlich, unumkehrbar und ungeheuer schnell“ [8, 9].

Die Digitalisierung ist bereits dabei, mehr und mehr Lebensbereiche zu durchdringen, so auch das Berufs- und Arbeitsleben [10]. Daraus ergibt sich, dass für alle diese Bereiche konkrete Strategien benötigt werden, um Gelingensbedingungen für die „digitale Transformation“ zu schaffen [11]. Dementsprechend hat die Europäische Union (EU) 2021 mit dem Aufbauplan *NextGenerationEU* im Zuge der *Fit-for-55-Strategie* eine „digitale Dekade“ ausgerufen, um Ziele wie die Vermittlung digitaler Kompetenzen zu erreichen [12], wobei „digitale Kompetenzen“ zu den 8 Schlüsselkompetenzen lebenslangen Lernens [13] zählen, die in einem gemeinsamen Referenzrahmen (DigiComp; [14–16]) genauer beschrieben sind. Als Antwort auf die im Europäischen Qualifikationsrahmen (EQR) gemachten Vorschläge zum Erwerb von erforderlichen digitalen Kompetenzen [14, 17] hat die Bundesregierung eine konkrete Digitalisierungsstrategie ausgearbeitet, die im Bildungsbereich Maßnahmen wie die *Berufsbildung 4.0 *umfassen. Letzteres unterstreicht eindrücklich die Relevanz eines Wissenstransfers digitaler Kompetenzen für die Aus‑, Fort- und Weiterbildung. Dies betrifft auch die Gesundheitsberufe.

Die digitale Transformation der Gesundheitsversorgung wirkt sich auf das Berufsbild der Pflegefachpersonen insofern aus, dass im wissenschaftlichen Diskurs kaum noch von einer Ersetzung der Pflegefachpersonen durch digitale Technologien die Rede ist [18–20], sondern zunehmend von der Herausbildung einer „komplementären Beziehung“ [21, 22]. Auch die Angst vor dem „Ersetztwerden“ weicht damit sukzessive der Erkenntnis einer sinnvollen Ergänzung und Unterstützung [23, 24]. Andere Stimmen hingegen beschreiben die Gefahr der Professionalisierung bei gleichzeitiger Deprofessionalisierung, wenn beispielsweise die körperlich-leibliche und damit verbundene pflegetherapeutische Basis beruflichen Handelns geschmälert oder Rationalisierungsbestrebungen gefördert würden [18, 19, 25–27]. Diese 2 Seiten des Diskurses versinnbildlichen die Notwendigkeit eines „digitalen Kompetenzerwerbs“, da für eine kompetente und somit auch reflektierte Handhabung digitaler Technologien die Qualifikation und der Kompetenzaufbau der Pflegefachpersonen für die zielführende Nutzung von besonderer Relevanz sind [28–32]. Zwar ist die Nutzung digitaler Instrumente in Pflegeeinrichtungen bereits seit mehreren Dekaden üblich. Die Einführung der ersten Krankenhausinformationssysteme (KIS) beispielsweise geht bereits auf die 1970er-Jahre zurück [25, 33, 34]. Damit beschreibt der Begriff der „Digitalisierung“ jedoch lediglich die digitale Repräsentation analoger Inhalte mittels eines technischen Tools und die Übersetzung von analogen Werten in Form von Bits und Bytes [22, 35, 36]. Darüber hinausgehend umfasst die „digitale Transformation“ allerdings auch die Anbahnung einer zunehmenden Vernetzung, freien Informationsfluss und normative Erwartungen von Partizipation, Transparenz und Authentizität durch digitale Applikationen [22].

Die digitale Transformation beinhaltet also auch Veränderungsprozesse in Hinblick auf das Selbstbild, Werte und Haltungen [22, 36]. Sie geht damit weit über die EDV-basierte Erfassung von Patientendaten hinaus. Diesbezügliche Anwendungskompetenzen sollten in der Ausbildung erworben werden [25] sowie das Wissen um die Passgenauigkeit neuer Technologien zu versorgungsrelevanten Problemen. Kompetenzen sind damit beispielsweise auch im Bereich der ethischen und rechtlichen Reflexion zu erwerben. Gerade hier haben die Gesundheitsberufe, allen voran die Pflegeberufe, traditionell die Aufgabe, ethische Diskussionen anzustoßen, wozu die notwendige reflexive Haltung systematisch zu erwerben ist.

Soweit hier und im weiteren Verlauf über den Erwerb von Digitalkompetenz(-en) gesprochen wird, ist daher von einem Set von Fähigkeiten (Umgang mit komplexen Informationen und Daten, Kommunikation mit digitalen Werkzeugen und kollaboratives Arbeiten, Produktion digitaler Inhalte, sichere Nutzung digitaler Endgeräte und Dienste sowie grundlegende Problemlösungsfähigkeit in einer fluiden, digitalen Umwelt) die Rede [14, 16]. Der Erwerb von digitalen Kompetenzen für die Gesundheits- und Pflegefachberufe, für die bereits erste Vorschläge vorliegen [37–41], eröffnet die Chance eines gemeinsamen Reflexionsprozesses und die Erfahrung, dass digitale und assistive Technologien sowie ihre Einbindung in Arbeitsprozesse gestaltbar und keineswegs naturwüchsig sind [20]. Digitale Kompetenz im eigentlichen Sinne umfasst neben dem Anwendungswissen (Digital Skills) auch die Befähigung zur Abschätzung der Folgen, die sich für Einzelne und die Gesellschaft [42] aus der Anwendung digitaler und assistiver Gesundheitstechnologien ergeben.

Bildungseinrichtungen wie Fach- und Berufsschulen haben unter anderem die Aufgabe, die Inhalte von Aus‑, Fort- und Weiterbildung orientiert an den Anforderungen eines zukünftigen Arbeitsmarktes auszugestalten. Für das Berufsfeld der Pflegefachpersonen sollte die Ausbildung beispielsweise Wissen im Umgang mit elektronischer Dokumentation oder auch Robotik und Telemedizin bzw. Telepflege beinhalten sowie in diesem Zusammenhang auch die Zusammenarbeit in interprofessionell agierenden Teams sowie entsprechende Kompetenzanforderungen und Rollenverhältnisse vermitteln.

Ein erster Schritt in Richtung einer Modernisierung war die Novellierung des Pflegeberufegesetzes (PflBG) im Jahr 2017 und die daraus resultierende *generalistische Pflegeausbildung*. Die Reorganisation der Pflegeausbildung beabsichtigte die qualitative Verbesserung der Ausbildung durch neue Lerninhalte und mehr Praxisanleitung sowie die Kompetenzvermittlung für ein breites Spektrum pflegerischer Aufgaben, die zur professionellen Pflege von Menschen aller Altersstufen in akut und dauerhaft stationären sowie ambulanten Pflegesituationen erforderlich sind [43]. Dazu wird unter Berücksichtigung des reformierten Pflegeberufegesetzes zwischen selbstständig, eigenständig und interdisziplinär auszuführenden Aufgaben unterschieden [43]. Zur Zielerreichung wurde von der Bundesregierung eine Fachkommission eingesetzt, die einen Rahmenlehrplan für den theoretischen und praktischen Unterricht und einen Rahmenausbildungsplan für die praktische Pflegeausbildung entwickeln sollte. Im Jahr 2019 wurden diese „Rahmenpläne der Fachkommission nach § 53 PflBG“ mit empfehlender Wirkung veröffentlicht [44]. Bei der Erstellung der neuen Rahmenpläne gab es die Möglichkeit, notwendige Lerninhalte im Bereich digitaler assistiver Technologien (DAT) aufzugreifen und Vorschläge zu machen, um die Berufsausbildung auch in diesem Punkt zukunftsfähig zu gestalten.

Die neu gestalteten Rahmenpläne als Regelwerk für die theoretische Ausgestaltung der Ausbildung dient den Pflegeschulen und den Trägern der praktischen Ausbildung als Orientierungshilfe für die notwendige Entwicklung der schulinternen Curricula und der Ausbildungspläne. Auch für die Länder stellen sie eine Orientierung dar, ohne jedoch in deren Durchführungszuständigkeit einzugreifen [44].

Diese Arbeit geht der Frage nach, inwieweit die Rahmenpläne der Fachkommission (im Folgenden als „Bundesrahmenplan“ bezeichnet) und in der Folge die Länderrahmenpläne^1^ im Zuge der Novellierung der Pflegeberufeausbildung auf die Anforderungen der digitalen Transformation reagieren. Wird in den Länderrahmenplänen ein digitaler Kompetenzerwerb adressiert? Es werden die Lehr- und Lernkontexte aus den einzelnen Länderrahmenplänen extrahiert, in die Aspekte der Digitalisierung eingebettet sind. Ebenfalls wird untersucht, welche Versorgungskontexte hinsichtlich welcher bereits verfügbaren Technologien angesprochen sind. Es ist von Interesse zu erfahren, inwieweit mit den Rahmenplänen ein Anreiz zur Einbettung digitaler Themen in praxisrelevante Felder gesetzt wird.

## Material und Methoden

Die Analyse der Rahmenpläne fand zwischen August und Oktober 2021 statt. Mittels Sichtung der offiziellen Webseiten der Bundes- und Landesministerien wurde auf die Länderrahmenpläne zugegriffen. Im Anschluss an eine erste Sichtung wurden die zuständigen Organisationseinheiten in den jeweiligen Bundes- und Landesbehörden zur Verifizierung hinsichtlich der Aktualität der veröffentlichten Länderrahmenpläne kontaktiert.

Als Studiendesign wurde eine explikativ-qualitative Inhaltsanalyse gewählt [45]. Das Vorgehen ist für eine komparative Analyse von zuvor als interpretationsbedürftig identifizierten Bedeutungsinhalten von Textmaterial geeignet, da das Textmaterial systematisch in ein Verhältnis zu Kontextinformationen gesetzt wird [46]. Es wird durch einen kontextbeschreibenden Vergleich im Datenmaterial möglich, kategoriale Zusammenhänge [47] zwischen dem Themenfeld „Digitalisierung“, seiner „methodisch-didaktischen Bezugnahme“ sowie den pflegerischen Handlungsfeldern innerhalb der Rahmenpläne offenzulegen. Da die Reichweite des Kontextes durch die Themenstellung und das Material begrenzt war, wurde im Sinne einer engen Kontextanalyse vorgegangen [45]. Für die enge Kontextanalyse wurden Informationen aus demselben Material herangezogen, aus dem auch die interpretationsbedürftigen Textstellen stammten [45].

Zur Eingrenzung und auf Basis eines informellen Peer-Group-Konsensverfahrens [48] wurden 3 das Metathema „Digitalisierung“ kennzeichnende Schlagworte festgelegt: „digital*“, „assistenz*“ und „tele*“. In einem ersten Analyseschritt wurden sowohl der Bundesrahmenplan als auch die Länderrahmenpläne unter Zuhilfenahme der trunkierten Wortsuchfunktion „Sublime 3“ in der Version „Build 3211“ nach entsprechenden Textstellen durchsucht und die Funde in einer Tabelle zur Informationsvisualisierung festgehalten [49]. Die eigentlichen Daten für die Analyse bestehen damit aus den Fundstellen der 3 vorher festgelegten Schlagworte sowie ihrer kontextuellen Einbettung innerhalb der vorliegenden Länderrahmenpläne. Das Vorgehen ermöglichte eine erste quantitative Einschätzung hinsichtlich einer Anhäufung der kategorialen Schlagwörter innerhalb der einzelnen Textkorpora. Nach Abschluss dieser Vorarbeiten wurden die Texte einer erneuten „Sublime-3“-Kontextanalyse unterzogen. Durch den somit möglichen und auf dem Prinzip der „gleichzeitigen Variation“ beruhenden Textvergleich war die Bildung von relativen Häufigkeiten der gebräuchlichen Schlagwörter möglich. Der durch diese Analyseform eröffnete kontextuelle Zugang zu intratextuellen Stichwortverbindungen ließ im Sinne einer gefassten strukturellen Textanalyse eine stringente Beschreibung von textinternen Bezügen (des „Co-Textes“) zu und legte damit die Beziehungen von Textteilen zum fokussierten Untersuchungsgegenstand (Rolle der digitalen Kompetenzen) offen [50]. Somit konnte auch die strukturelle Einbindung von Kontextfaktoren, die die trunkierten Schlagworte beinhalteten (u. a. Unterrichtsumfang, Zeitpunkt oder Lernfeldzuordnung), identifiziert und für die Analyse sichtbar gemacht werden [51, 52].

Die Stichwortverbindungen und dazugehörigen Textabschnitte aus den Länderrahmenplänen wurden in einer Matrix festgehalten und durch einen auf dem Peer-Group-Prinzip beruhenden Qualitätssicherungsprozess durch die Autor:innen auf verschiedene Merkmale hin untersucht [45]. Die durch dieses Vorgehen letztendlich identifizierten Materialstellen wurden in einem letzten Schritt vertiefend inhaltsanalytisch aufbereitet und anhand einschlägiger Ankerbeispiele exemplarisch beschrieben. Ankerbeispiele verdeutlichen anhand der konkreten Textstellen beispielhaft die identifizierten Merkmale im Textmaterial. Damit war eine abschließende, (induktiv-)interpretative kategoriale Darstellung hinsichtlich der durch die diversen Länderrahmenpläne empfohlenen Integration von Digitalisierungsaspekten im Gesamtkontext der generalistischen Ausbildung zur Pflegefachperson möglich [53, 54]. Alle Analyse- und Interpretationsschritte wurden durch ein stetiges Peer-Group-Monitoring qualitätssichernd durchgeführt [46].

## Ergebnisse

Es zeigte sich, dass lediglich die Länder Bremen, Sachsen, Sachsen-Anhalt, Baden-Württemberg, Berlin und Brandenburg zum Untersuchungszeitpunkt einen landesspezifischen Rahmenplan für die generalistische Pflegeausbildung veröffentlicht hatten. Die Bundesländer Berlin und Brandenburg greifen auf einen gemeinsamen Landesrahmenplan zu. Die restlichen Bundesländer Bayern, Thüringen, Mecklenburg-Vorpommern, Schleswig-Holstein, Hamburg, Nordrhein-Westfalen, Rheinland-Pfalz, Saarland, Niedersachsen und Hessen orientieren sich inhaltlich vollständig am Bundesrahmenplan und haben keine expliziten Veränderungen, Vertiefungen oder Anpassungen vorgenommen. Somit konnten insgesamt 6 Entwürfe für bundeslandspezifische Rahmenlehrpläne inhaltlich untersucht werden.

Die Schlagwortanalyse erbrachte, dass der Bundesrahmenplan an 55 Textstellen das Themenfeld „digital*“ sowie an 17 Textstellen das Themenfeld „assistenz*“ aufgreift (Tab. 1). Keine Treffer ergaben sich für das Themenfeld „tele*“. Die eingeschlossenen Länderrahmenpläne wiesen hinsichtlich der Häufigkeiten des Vorkommens der eingeschlossenen Schlagwörter eine unterschiedliche Spannweite auf. Beispielsweise konnte das Schlagwort „digital*“ im Landesrahmenplan von Berlin und Brandenburg mit 7 Treffern und im Rahmenplan des Landes Sachsen-Anhalt mit 86 Treffern identifiziert werden. Das Schlagwort „assistenz*“ war im Rahmenplan von Berlin und Brandenburg lediglich mit 4 Treffern, aber im Rahmenplan des Landes Sachsen-Anhalt mit 21 Fundstellen nachweisbar. Das Schlagwort „tele*“ war ausschließlich im Länderrahmenplan des Landes Sachsen-Anhalt mit einem Treffer nachweisbar. Die vollständige Darstellung der Häufigkeiten der identifizierten Schlagworte ist in Tab. 1 dargestellt.

**Table 1:** 

Bundesland			
	Digital (digital*)	Assistenzsystem (assistenz*)	Telepflege/Telepräsenzsysteme (tele*)
Berlin und Brandenburg	7	4	0
Sachsen	59	17	0
Sachsen-Anhalt	86	21	1
Bremen	27	6	0
Baden-Württemberg	40	13	0
Bundesrahmenplan (restl. Bundesländer)	55	17	0

Die Länderrahmenpläne zeigten in der Kontextanalyse lediglich eine punktuelle Bezugnahme auf „Digitalisierung“ als Qualifizierungsaspekt der neuen generalistischen Ausbildung. Es waren keine zusammenhängenden Text- oder Themenblöcke nachweisbar, die entsprechende Themen als essenziellen Bestandteil von inhaltlichen Ausführungen, wie z. B. von Pflegeprozessen, beschreiben. Durch eine tabellarische Darstellung ließen sich die zuvor im Text systematisch identifizierten Textstellen als Ankerbeispiele den entsprechenden Suchwörtern zuschreiben. Die vertiefende und differenzierte Beschreibung der kontextuellen Zusammenhänge der identifizierten Textstellen, u. a. zu den Bereichen „adressierte Kompetenzen“ und „Kompetenzzuordnung Ausbildungs- und Prüfungsverordnung für die Pflegeberufe“ (PfAPrV) sowie „Umfang von Unterrichtseinheiten“ (UE) innerhalb der verschiedenen Länderrahmenpläne kann in einer Übersichtstabelle (s. Onlinematerial, Tabelle Z1) eingesehen und nachvollzogen werden.

Es zeigte sich, dass der Begriff „digital*“ in 2 grundsätzlichen Kontexten vorkommt: in der methodisch-didaktischen Ausrichtung sowie im Kontext der Praxisimplementierung (Tab. 2). In Bezug auf die methodisch-didaktische Ausrichtung wurden vor allem die Art und Weise und das Format beschrieben, in dem digitale Elemente in Lehr- und Lernsituationen zum Einsatz kommen sollen. Beispielhaft seien die Integration einer „digitalen Schnitzeljagd“ unter Zuhilfenahme von Methoden aus dem Bereich der Gamification (VR-Brillen) genannt. Die Begriffe „digital*“ und „assistenz*“ wurden in den meisten Länderrahmenplänen im gleichen Kontext genannt und werden eher allgemein beschrieben („… Nutzung technischer und digitaler Assistenzsysteme“).

**Table 2:** 

Bundesland				
	Methodik (didaktische Einbettung)	Digital (digital*) Praxisimplementierung (Inhalt)	Assistenzsystem (assistenz*)	Telepflege/Telepräsenzsysteme (tele*)
Berlin u. Brandenburg	… digitale Schnitzeljagd über das Schulgelände, den Einsatz einer VR-Brille zum Erklären der Atemorgane und eine Stop-Motion-Animation zur Durchführung der Pflegevisite (S. 78)	Nutzen analoge und digitale Pflegedokumentationssysteme, um ihre Pflegeprozessentscheidungen in der Pflege von Menschen aller Altersstufen selbstständig und im Pflegeteam zu evaluieren (S. 55)	Reflexion des Einbezugs technischer Assistenzsysteme in unterschiedlichen Pflege- und Betreuungskontexten sowie der Potenziale und Grenzen (S. 61)	–
Sachsen	Erkundungsaufgabe zu ausgewählten aktuellen spezifischen technischen und digitalen Assistenzsystemen in stationären bzw. teilstationären Einrichtungen … (S. 146)	Technische/digitale Hilfsmittel für gesundheitsförderliche/präventive Informations- und Beratungsangebote nutzen (z. B. Gesundheits-Apps/Telecare etc.) und kritische fachliche Reflexion der Angebote (S. 63)	Beobachtungs- und Reflexionsaufgabe einer Schulung im Umgang mit ausgewählten technischen und digitalen Assistenzsystemen (S. 137)	–
Sachsen-Anhalt	Schulführung durch Azubis des 2. Ausbildungsjahres oder „Schulrallye“ mit digitalen Medien (S. 33)	Nutzung digitaler Hilfen zur Informationserhebung, -dokumentation und -auswertung für die Bewegung (S. 25)	Erkundung digitaler Assistenzsysteme (S. 72)	Exkursion zur Praxiserprobung im Skills- und FutureCareLab an den Universitäten Halle und Magdeburg: „digitale Gesundheitsversorgung“ (Beratung anhand eines Telepräsenzsystems, digitale Hilfsmittel, Robotik, Telemonitoring; S. 59)
Bremen	–	Menschen bei Alltagsaktivitäten in ihrer Mobilität unterstützen und bei Bedarf technische und digitale Hilfsmittel nutzen (S. 45)	Durchführung von gezielten Schulungen zur Förderung der Alltagsbewältigung unter Berücksichtigung biografisch bedingter Gewohnheiten, von Lebenslagen und sozialen Unterstützungssystemen sowie unter Nutzung technischer und digitaler Assistenzsysteme (S. 135)	–
Baden-Württemberg	Erkundung bzw. Exkursion hinsichtlich situativ geeigneter technischer und digitaler Assistenzsysteme (z. B. Exoskelett, Sprachcomputer; S. 104)	Vergleichende Erhebung zum Einsatz von technischen und digitalen Hilfsmitteln in der Entwicklung, Förderung und Erhaltung von Bewegungsfähigkeit (S. 16)	Informationsbedarfe zu technischen und digitalen Assistenzsystemen (z. B. Sprachcomputer; S. 110)	–
Bundesrahmenplan (restl. Bundesländer)	Erkundungsaufgabe zu ausgewählten aktuellen spezifischen technischen und digitalen Assistenzsystemen in stationären bzw. teilstationären Einrichtungen … (S. 121)	Technische und digitale Hilfsmittel zur Unterstützung bei der Bewegungsförderung und Positionierung sowie Regelungen zu deren Verfügbarkeit (z. B. Medizinproduktegesetz; S. 39)	Schulung zu technischen Assistenzsystemen (S. 126)	–

Im Bundesrahmenplan, der von 10 Bundesländern anstelle eines eigenen Landesrahmenplans genutzt wurde, fehlten konkrete Handlungsempfehlungen hinsichtlich situativ geeigneter Anwendungsbeispiele für digitale Anwendungen fast vollständig. Es waren nur wenige detaillierte Ausbildungsangebote auffindbar, worin sich die Länderrahmenpläne vom Bundesrahmenplan unterscheiden.

Auch Anregungen für den praktischen Teil der Ausbildung erfolgen im Bundesrahmenplan eher unspezifisch: „Erkundungsaufgaben zu ausgewählten aktuellen spezifischen technischen und digitalen Assistenzsystemen in stationären bzw. teilstationären Einrichtungen“ (Tab. 2). In den Länderrahmenplänen dagegen werden konkrete Vorschläge gemacht, wie z. B. eine Exkursion zur Praxiserprobung technischer und digitaler Assistenzsysteme im Umfeld eines Reallabores (Skills-Lab) in Baden-Württemberg.

Die 3 Länderrahmenpläne von Berlin und Brandenburg, Bremen und Baden-Württemberg zeigten insgesamt geringere Treffer hinsichtlich der gesuchten Schlagworte verglichen mit dem Bundesrahmenplan (Tab. 1). Die Kontextanalyse ergab jedoch, dass diese geringere Dichte an thematischen Schlagworten keine inhaltliche Schlechterstellung hinsichtlich der Kontextualisierung der Themenfelder bedeutet. Vielmehr wiesen die qualitativen Ausführungen einen ähnlichen Detaillierungs- und Kontextualisierungsgrad wie in den Länderrahmenplänen von Sachsen und Sachsen-Anhalt auf, bei denen sich eine höhere Anzahl von Schlagworttreffern ergab.

Lediglich in Baden-Württemberg und Sachsen-Anhalt wurden konkrete Handlungsempfehlungen für Ausgestaltungsmöglichkeiten von praktischen Übungsformaten und beispielsweise Exkursionen in Reallabore empfohlen (Tab. 2). Bei Reallaboren handelt es sich um Angebote zweier universitärer Einrichtungen, die die notwendigen Ressourcen vorhalten, um Fachschulen konkret im Theorie-Praxis-Transfer zu unterstützen.

Das Schlagwort „tele*“ fand sich lediglich im Länderrahmenplan von Sachsen-Anhalt (Tab. 1). Zur Kompetenzvermittlung im Umgang mit „Telepräsenzsystemen“ wird ihre didaktische Einbettung in konkrete Handlungsszenarien empfohlen und z. B. die Nutzung als digitales Hilfsmittel in Bereichen wie „Beratung“ vorgeschlagen (Tab. 2).

## Diskussion

Im Bundesrahmenplan wie auch in den Länderrahmenplänen sind erste Hinweise auf die Relevanz des Erwerbs von Anwendungskompetenzen hinsichtlich digitaler assistiver Technologien (DAT) angelegt. Insbesondere in den Länderrahmenplänen der beiden Bundesländer Baden-Württemberg und Sachsen-Anhalt sind Ansätze enthalten, das Thema „Digitalisierung“ zielgerichtet zu positionieren und digitale Kompetenzen theoretisch aufbereitet in die praktische Ausbildung einfließen zu lassen. Ein zentraler Aspekt ist in diesen beiden Länderrahmenplänen der praxisorientierte Kompetenzaufbau. Der Kompetenzerwerb im Sinne eines konkreten Theorie-Praxis-Transfers soll dabei idealerweise in einem Reallabor umgesetzt werden. Dort nicht enthalten waren weiterführende didaktische und methodische Verweise zur Implementierung von DAT (z. B. szenarienbasierte Handlungsempfehlungen), um auch Lehrenden das notwendige Rüstzeug für die Umsetzung der curricularen Vorschläge an die Hand zu geben. Dies erscheint jedoch ebenfalls vor dem Hintergrund wertvoll, als dass aktuelle Konzepte der Berufsbildung eine fachsystematisch aufeinander aufbauende Wissensvermittlung längst verlassen haben und Wissen sowie Können eher im Kontext von handlungs- und situationsorientierten Kompetenzen entwickelt werden [43].

Der Bundesrahmenplan skizziert 4 grundlegende Kompetenzbereiche, die den Megatrend „Digitalisierung“ aufgreifen [44, 55]. Diese Kompetenzbereiche sind: „Informations- und Datenkompetenz“, „Kommunikation und Zusammenarbeit“, „digitale Inhalte und Erstellung“, „Problemlösung und Sicherheit“. Konkrete Beispiele bezüglich der Inhalte dieser Kompetenzbereiche bzw. Hinweise für darauf aufbauende Implementierungskonzepte sind im Bundesrahmenplan nicht auffindbar. Zur Vermittlung konkreter Kompetenzen werden keine Angaben gemacht. Weiterführende Empfehlungen und Vorschläge zur Ausgestaltung entsprechender Kompetenzbereiche oder gar die praxisbezogene Integration in die berufliche Ausbildung erfolgten ebenfalls nicht.

Durch die Lücken im Bundesrahmenplan und in den Rahmenplänen der Länder ergeben sich für die Pflegeschulen Schwierigkeiten und Unsicherheiten, ihre Ausbildungsangebote an die Anforderungen eines zukünftigen Arbeitsmarktes und damit auch an die entsprechenden Bedarfe hinsichtlich eines digitalen Kompetenzerwerbs der Auszubildenden anzupassen. Die allgemein fehlende Definition von „Digitalisierung“ im Kontext Gesundheits- und Pflegeversorgung sowie die fehlende Präzisierung von Ausbildungsanforderungen und -inhalten sind einer zukunftsfähigen Ausgestaltung der Pflegeberufe abträglich. Es fehlen konkrete Formulierungen zum Wissenserwerb, beispielsweise zum Umgang mit elektronischer Dokumentation, Telemedizin und robotischen Assistenzsystemen, aber auch zur ethischen, normativen und rechtlichen Reflexion. Das heißt, dass die für eine effektiv entlastende und zugleich kritisch-konstruktive Nutzung digitaler Anwendungen in der Versorgungspraxis erforderlichen Kompetenzen insgesamt nur bedingt Berücksichtigung finden und es den Schulen überlassen bleibt, entsprechende Inhalte nach eigenen Vorstellungen auszugestalten [35].

Digitale Kompetenzen werden also anscheinend kaum als Querschnittskompetenz betrachtet und somit nicht systematisch in die Rahmenpläne implementiert. Eine Anpassung des Bundesrahmenplans und der Länderrahmenpläne ist notwendig, um die identifizierten Lücken zu schließen und zukünftig einen mehrwertschaffenden und anwendungsorientierten Theorie-Praxis-Transfer zu erreichen. Digitale Kompetenzen sollten zum Beispiel in Anlehnung an das Strategiepapier „Bildung in der digitalen Welt“ der Kultusministerkonferenz [56] konkreter definiert und exemplifiziert werden.

Generell werden für eine gelingende digitale Transformation der Gesundheitsberufe allgemein und speziell für die Vermittlung digitalisierungsbezogener Kompetenzen im Rahmen von Aus‑, Fort- und Weiterbildung übergeordnete Prinzipien und/oder Ordnungskategorien benötigt. In diesem Punkt könnte die Fachkommission Hinweis- und Ratgeber sein, indem sie externe, interne und soziale Evidenz zur digitalen Transformation der Gesundheitsversorgung für eine zukunftsorientierte Ausbildung der Pflegefachberufe aufbereitet, zur Verfügung stellt und in die Rahmenpläne einfließen lässt. Damit könnte auch der Zugzwang auf die Länder erhöht werden, Digitalisierungsaspekte in den Länderrahmenplänen umfassender zu berücksichtigen und diese idealerweise auch länderübergreifend zu vereinheitlichen.

### Limitationen

Es kann nicht gänzlich ausgeschlossen werden, dass einzelne Bundesländer während der vorliegenden Analyse weitere Ergänzungen in den Länderrahmenplänen vorgenommen haben. Zudem ist auf die im PflBG vorgeschriebene Pflicht für Pflegeschulen zur Vorlage eines schuleigenen Curriculums hinzuweisen. Die Wirkung des Bundesrahmenplans und der Länderrahmenpläne wird dadurch relativiert, woraus sich für die Schulen Freiräume für die inhaltliche Gestaltung der internen Curricula ergeben. Gleichermaßen ließen sich Implikationen für die Studiengänge zur Ausbildung des Lehrpersonals sowie für gezielte Weiterbildungsangebote zur Umsetzung des neuen PflBG ableiten. Eine weitere Limitation ist in diesem Zusammenhang, dass im Rahmen dieser Untersuchung nicht danach gefragt werden konnte, inwieweit das Lehrpersonal im Unterricht eigenständig Digitalisierungsaspekte und -kompetenzen vermittelt. Dies könnte Gegenstand weiterführender und vertiefender Studien sein.

## Fazit

Trotz der Relevanz des Themenkomplexes „Digitalisierung“ und obwohl die Länderrahmenpläne hinsichtlich der Verwendung der auf Digitalisierungsinhalte hindeutenden Schlagworte eine relative Nähe zum Bundesrahmenplan aufweisen, zeigt die inhaltliche Auseinandersetzung keine methodisch-didaktische Verankerung dieses Themenfeldes in den Rahmenplänen insgesamt. Somit ist auch eine Umsetzung in den Schulen nur bedingt möglich. Es wird daher dringend empfohlen, den Bundesrahmenplan bis zum Ende des Nachbesserungszeitraums im Jahr 2024 diesbezüglich noch anzupassen (siehe auch Infobox 1 zu den Kernaussagen).

Es wäre möglich, das Thema „Digitalisierung“ systematisch und kompetenzbasiert zu verankern, nicht zuletzt auch, um die professionelle Weiterentwicklung der Pflegeberufe im Sinne einer ressourcen- sowie zukunftsorientierten und optimierten Pflegepraxis zu unterstützen. Der Aufbau von digitalen Kompetenzen sowie der Erwerb von Anwendungswissen zu digitalen und assistiven Technologien bereits in der Ausbildung bilden das Fundament für das zukünftige Berufsleben. Auf akademischem Niveau stellt der Erwerb dieser Kompetenzen einen wichtigen Schritt zur (akademischen) Professionalisierung der Pflege dar, wodurch auch Pflegekräfte die Möglichkeit bekommen, die digitale Transformation der Gesundheitsversorgung selbst mitzugestalten. Zu empfehlen sind aus diesem Grund eine engere Verzahnung mit hochschulischen Kooperationspartnern (u. a. Reallaborausbildung) sowie der Ausbau akademischer Strukturen durch die Erweiterung von evidenzbasierten Weiterbildungsangeboten für Lehrpersonal im Bereich der Digitalisierung. Wünschenswert wäre in Bezug auf den Wissenstransfer die Festlegung eines erforderlichen Mindestumfangs über den gesamten Ausbildungsverlauf hinweg. Die Abgrenzung von akademischen und nichtakademischen Ausbildungsinhalten erscheint dabei sinnvoll, um unterschiedliche Kompetenzniveaus des Europäischen (EQR) und des Deutschen Qualifikationsrahmens (DQR) aufzugreifen.

### Infobox 1 Kernaussagen

- Die Digitalisierung erfordert veränderte Kompetenz- und Rollenprofile der Pflegefachberufe, damit ein fachgerechter Umgang mit digitalen und assistiven Technologien (DAT) gewährleistet ist.
- Die „Rahmenpläne der Fachkommission nach § 53 PflBG“ von 2019 weisen kaum Bezüge zum Aufbau erforderlicher digitaler Kompetenzen auf.
- 2 Länderrahmenpläne (aus Baden-Württemberg und Sachsen-Anhalt) konkretisieren Vorschläge zum Aufbau digitaler Kompetenzen für konkrete Pflegesituationen.
- Eine Nachbesserung der Länderrahmenpläne hinsichtlich der Integration digitaler Kompetenzen wird empfohlen.

## Supplementary Information





## Footnotes

1 Da die Betitelung der Rahmenpläne innerhalb der Länder uneinheitlich ist, wird im Folgenden bei Bezugnahme auf die Länderpläne für eine bessere Verständlichkeit und Lesbarkeit von „Länderrahmenplänen“ gesprochen.
