# Supplementary material for: Teaching digital competences in nursing education—a comparative analysis of the federal and state framework curricula
Source: Bundesgesundheitsblatt Gesundheitsforschung Gesundheitsschutz. 2022 Aug 9;65(9):891–9. [Article in German] doi: 10.1007/s00103-022-03575-2 (PMC9436847; doi:10.1007/s00103-022-03575-2)
Supplement: Supplementary file 1 [file 103_2022_3575_MOESM1_ESM.pdf]

Bundesgesundheitsblatt – Gesundheitsforschung – Gesundheitsschutz, Ausgabe 9/2022

Onlinematerial zum Beitrag:

Vermittlung digitaler Kompetenzen in der Pflegeausbildung – eine Vergleichsanalyse der Rahmenpläne von Bund und Ländern

Sebastian Hofstetter[1,2], Lisa Lehmann[2], Max Zilezinski[1,2], Jenny-Victoria Steindorff[1], Patrick Jahn[1,2], Denny Paulicke[1,3]

ORCID-iDs: Sebastian Hofstetter 0000-0003-3110-2379, Max Zilezinski 0000-0001-9225-1672, Patrick Jahn 0000-0002-1533-6717

[1] Universitätsmedizin Halle (Saale), AG Versorgungsforschung| Pflege im Krankenhaus, Department für Innere Medizin, Medizinische Fakultät, Martin-Luther-Universität Halle-Wittenberg, Halle (Saale), Deutschland

[2] Medizinische Fakultät, Martin-Luther-Universität Halle-Wittenberg, Digital HealthCare Hub, Dorothea-Erxleben-Lernzentrum Halle (DELH), Halle (Saale), Deutschland

[3] Akkon Hochschule für Humanwissenschaften, Fachbereich Medizinpädagogik, Berlin, Deutschland

Korrespondenzadresse:
Sebastian Hofstetter, M.A.
Medizinische Fakultät, Martin-Luther-Universität Halle-Wittenberg
AG Versorgungsforschung|Pflege im Krankenhaus
Universitätsmedizin Halle (Saale)
Ernst-Grube-Str. 40
06120 Halle (Saale)
Deutschland
Sebastian.Hofstetter@uk-halle.de

Tabelle Z1

Curriculare Einbindung des Themas Robotik/ Telepräsenzroboter in die Ausbildung zur Pflegefachfrau/ zum Pflegefachmann

AD = Ausbildungsdrittel

BL	AD	Kompetenz-zuordnung PflAPrV Anlage 2	Thema	Adressierte Kompetenzen	Inhalte	Umfang UE	Ergänzende Kommentierung/ Literaturverweise	Bezug Bundesrahmenlehrplan (Kernbereich)
1	3	IV 2b	Einführung in digitale Technologien/ Robotik	• kennen robotische Systeme zur Rehabilitation, zur Unterstützung des Personals und zur Unterstützung der Patienten/ Bewohner in unterschiedlichen Settings	• Roboter für Rehabilitation<br>• Roboter zur Unterstützung des Personals in Altenpflegeeinrichtungen, im Krankenhaus, in der ambulanten Pflege<br>• Roboter zur Unterstützung älterer und pflegebedürftiger Menschen zu Hause	4	Klein et al., (2018) *Robotik in der Gesundheitswirtschaft*, Heidelberg, medhochzwei Verlag GmbH	Umgang mit Pflegedokumentationssystem (analog / digital) (S. 165)
2	3	IV 2 c	Gesetzliche Grundlagen	• kennen gesetzliche Grundlagen zum Datenschutz, Schweigepflicht,<br>• E-Health-Gesetz, Fernbehandlungs-gesetz	• Datenschutz<br>• Schweigepflicht<br>• E- Health Gesetz<br>• Fernbehandlungsgesetz	2		Steuerung und Dokumentation pflegerischen Handelns (S. 202)
3	4	II 3b	ethische Einordnung	• kennen Empfehlungen des Ethikrates<br>• reflektieren ethische Konfliktsituationen im Zusammenhang mit Robotik	• Stellungnahme Robotik für gute Pflege<br>• Ethische Prinzipien, Fallarbeit	2<br><br>2	Deutscher Ethikrat, (2020) *Stellungnahme Robotik für gute Pflege,* Berlin	Steuerung und Dokumentation pflegerischen Handelns (S. 246)
4	4	V 1b, c	Telemedizin	• kennen evidenzbasierte Einsatzszenarien für telemedizinische Anwendungen<br>• kennen und finden Einsatzszenarien in ihrer beruflichen Lebenswelt	• Telekonsil<br>• Patientenmonitoring im häuslichen Umfeld<br>• Betrachtung unterschiedlicher Lebensbereiche (Klinik, Pflegeheim, Häuslichkeit)	4		Software für die Planung (S. 86)

5	5	IV 2 b<br>II 1 a	Telepräsenz-systeme	• kennen die Funktionsweise eines Telepräsenzroboters<br>• können einen TP bedienen<br>• kennen und bewerten die Kommunikation über einen TP<br>• leiten Schwerpunkte für das eigene Handeln ab	• Aufbau eines TP (exemplarisch)<br>• Funktionsweise<br>• Störungen und Lösungsmöglichkeiten<br>• Praktische Auseinandersetzung mit dem TP<br>• Praktische Übung<br>• Reflexion, Kriterien für die Kommunikation über einen TP	4		Steuerung und Dokumentation pflegerischen Handelns (S. 114)
	5	III 1a<br>III 3 a,b,c	Telepräsenz-system in der Televisite	• übernehmen Mitverantwortung Versorgung und Behandlung von Patienten, bringen ihre pflegefachliche Sichtweise in das Szenario ein,<br>• bearbeiten auftretende interprofessionelle Konflikte	• Interprofessionelles Training im Setting Televisite	6	Dorothea-Erxleben-Lernzentrum (Simulationspatienten; Future Care Lab, Digital Health Care Hub)	Pflegedokumentationssystem Informationen entnehmen (S. 118)
